# Supplementary material for: Maternal balanced energy-protein supplementation reshapes the maternal gut microbiome and enhances carbohydrate metabolism in infants: a randomized controlled trial
Source: Nat Commun. 2025 Mar 18;16:2683. doi: 10.1038/s41467-025-57838-y (PMC11920048; doi:10.1038/s41467-025-57838-y)
Supplement: Supplementary file 1 — Supplementary Information [file 41467_2025_57838_MOESM1_ESM.pdf]

Maternal balanced energy-protein supplementation reshapes the maternal gut microbiome and enhances carbohydrate metabolism in infants: a randomized controlled trial

Lishi Deng, Steff Taelman

Supplementary Information

Table S1: Baseline characteristics of MISAME-III participants and the subset participants

Characteristics	MISAME-III					Subset		
	Pre- and Postnatal Intervention (*n* = 475)	Prenatal Intervention (*n* = 462)	Postnatal Intervention (*n* = 471)	Control (*n* = 489)	*P* value	Pre-and Postnatal Intervention (*n* = 71)	Control (*n* = 81)	*P* value
**Health center catchment area, %**					0.99			0.99
Boni	23.6	22.1	22.1	23.5		15.5	13.4	
Dohoun	11.8	9.50	9.80	11.0		12.7	11.0	
Dougoumato II	18.5	17.5	18.3	18.6		22.5	15.9	
Karaba	9.9	10.6	10.2	10.0		12.7	12.2	
Kari	17.1	19.3	20.0	17.6		21.1	23.2	
Koumbia	19.2	21.0	19.7	19.2		15.5	24.4	
**Household level**								
Household food insecurity*, %	67.4	71.0	67.9	64.2	0.61	67.6	70.7	0.98
Wealth index, 0 to 10 points	4.62 ± 1.76	4.69 ± 1.73	4.55 ± 1.77	4.52 ± 1.77	0.45	4.70 ± 1.67	4.64 ± 1.87	0.17
**Maternal**								
Age, years	24.8 ± 6.24	25.1 ± 6.07	25.0 ± 6.33	25.1 ± 6.03	0.83	23.4 ± 5.56	25.2 ± 5.51	0.20
Ethnic group, %					0.31			0.37
Bwaba	55.6	59.1	57.5	56.9		49.3	53.7	
Mossi	35.2	33.8	35.0	35.6		39.4	32.9	
Others	9.26	7.14	7.22	7.57		11.3	13.4	
Religion of pregnant woman, %					0.44			0.91
Animist	20.8	23.8	23.6	22.9		22.5	22.0	
Muslim	42.9	41.6	42.0	42.3		47.9	43.9	
Catholic	12.4	13.6	15.1	13.9		7.04	6.10	
Protestant	21.3	16.9	15.7	16.8		19.7	25.6	
No religion, no animist	2.53	4.11	3.40	4.09		2.82	2.44	
Primary education and above, %	41.1	42.4	44.6	39.5	0.54	38.0	45.1	0.36
Number of jobs, %					0.68			0.33
0	61.7	60.0	61.6	59.7		64.8	53.7	
1	33.1	36.8	35.5	36.2		33.8	42.7	
2 and above	5.26	3.25	2.97	4.09		1.41	3.66	
Parity, %					0.18			0.41
0	24.2	22.5	23.8	20.0		29.6	20.7	
1-2	35.4	30.3	35.9	36.2		33.8	39.0	
3 or more	40.4	47.2	40.3	43.8		36.6	40.2	
Weight, kg	58.7 ± 8.88	58.0 ± 8.44	57.6 ± 8.24	58.4 ± 9.07	0.29	57.7 ± 8.30	60.2 ± 12.2	0.11
Height, cm	163 ± 5.75	163 ± 6.30	163 ± 5.88	162 ± 6.16	0.36	162 ± 4.96	163 ± 6.72	0.63
Body mass index, kg/m^2	22.1 ± 2.99	21.9 ± 2.73	21.8 ± 2.73	22.2 ± 3.00	0.13	22.0 ± 3.16	22.6 ± 3.74	0.16
Hemoglobin, g/dL	11.3 ± 1.48	11.2 ± 1.56	11.4 ± 1.53	11.4 ± 1.46	0.28	11.9 ± 1.38	11.6 ± 1.46	0.10
Anemia, hemoglobin <11 g/dL, %	38.5	41.6	38.0	36.8	0.49	26.8	34.1	0.06
Gestational age at birth, weeks	39.9 ± 1.71	40.1 ± 1.33	39.9 ± 1.63	39.7 ± 2.16	0.01	40.0 ± 1.63	39.6 ± 1.77	0.45
Gestational age at inclusion, weeks	11.6 ± 4.45	12.2 ± 4.50	11.8 ± 4.38	11.8 ± 4.61	0.24	9.61 ± 3.23	9.97 ± 3.73	0.79
**Intervention length when Sample collection, weeks**								
Mother – Tri2						13.3 ± 2.31	13.9± 2.43	0.51
Mother – Tri3						22.0 ± 3.49	21.5 ± 4.02	0.72
Mother – Pn12						39.3 ± 3.52	38.3 ± 4.56	0.46
Mother – Pn56						56.3 ± 3.35	55.6 ± 4.44	0.74
Infant – Pn12						39.5 ± 3.45	38.4 ± 4.38	0.40
Infant – Pn56						56.3 ± 3.42	55.7 ± 4.59	0.78

Values are percentages or means ± standard deviation s.

Group comparisons were conducted using ANOVA (two-sided) for continuous variables and chi-squared tests (teo-sided) for categorical variables.

*Assessed using FANTA/USAID's Household Food Insecurity Access Scale.

Table S2: Nutritional values of the BEP supplement for pregnant and lactating women[1].

	Mean for 72g (serving size)
Total energy (kcal)	393
Lipids (g)	26
Linoleic acid (g)	3.9
α-Linoleic acid (g)	1.3
Proteins (g)	14.5
Carbohydrates (g)	23.3
Calcium (mg)	500
Copper (mg)	1.3
Phosphorus (mg)	418
Iodine (µg)	250
Iron (mg)	22
Selenium (µg)	65
Manganese (mg)	2.1
Magnesium (mg)	73
Potassium (mg)	562
Zinc (mg)	15
Vitamin A (µg RE)[2]	770
Thiamin (mg)	1.4
Riboflavin (mg)	1.4
Niacin (mg)	15
Vitamin B5 (mg)	7
Vitamin B6 (mg)	1.9
Folic acid (µg)	400
Vitamin B12 (µg)	2.6
Vitamin C (mg)	100
Vitamin D (µg cholecalciferol)[3]	15
Vitamin E (mg α-tocopherol)[4]	18
Vitamin K (µg)	72

[1]Ingredients: vegetable oils (rapeseed, palm, soy in varying proportions), defatted soy flour, skimmed milk powder, peanuts, sugar, maltodextrin, soy protein isolate, vitamin and mineral complex, stabilizer (fully hydrogenated vegetable fat, mono and diglycerides).
[2]1 µg vitamin A RE = 3.333 IU vitamin A.
[3]1 µg cholecalciferol = 40 IU vitamin D.
[4]1 mg α-tocopherol = 2,22 IU vitamin E.
IU, international unit; RE, retinol equivalen

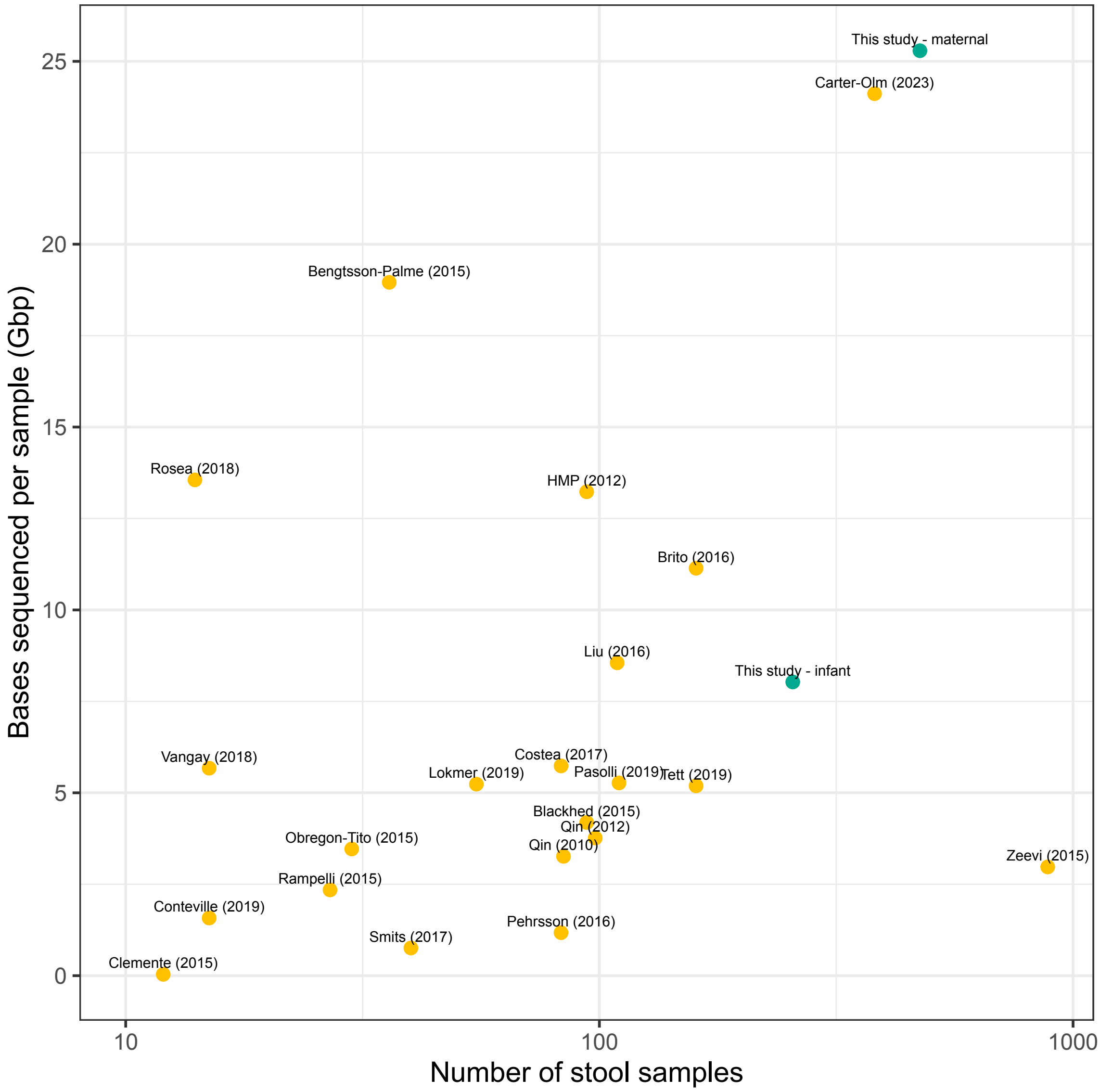

Figure S1: The number of samples versus the number of giga base-pairs (Gbp) sequenced per sample for other gut metagenomic datasets (yellow) and this study (green) [adapted from (Carter et al., 2023)[1]].

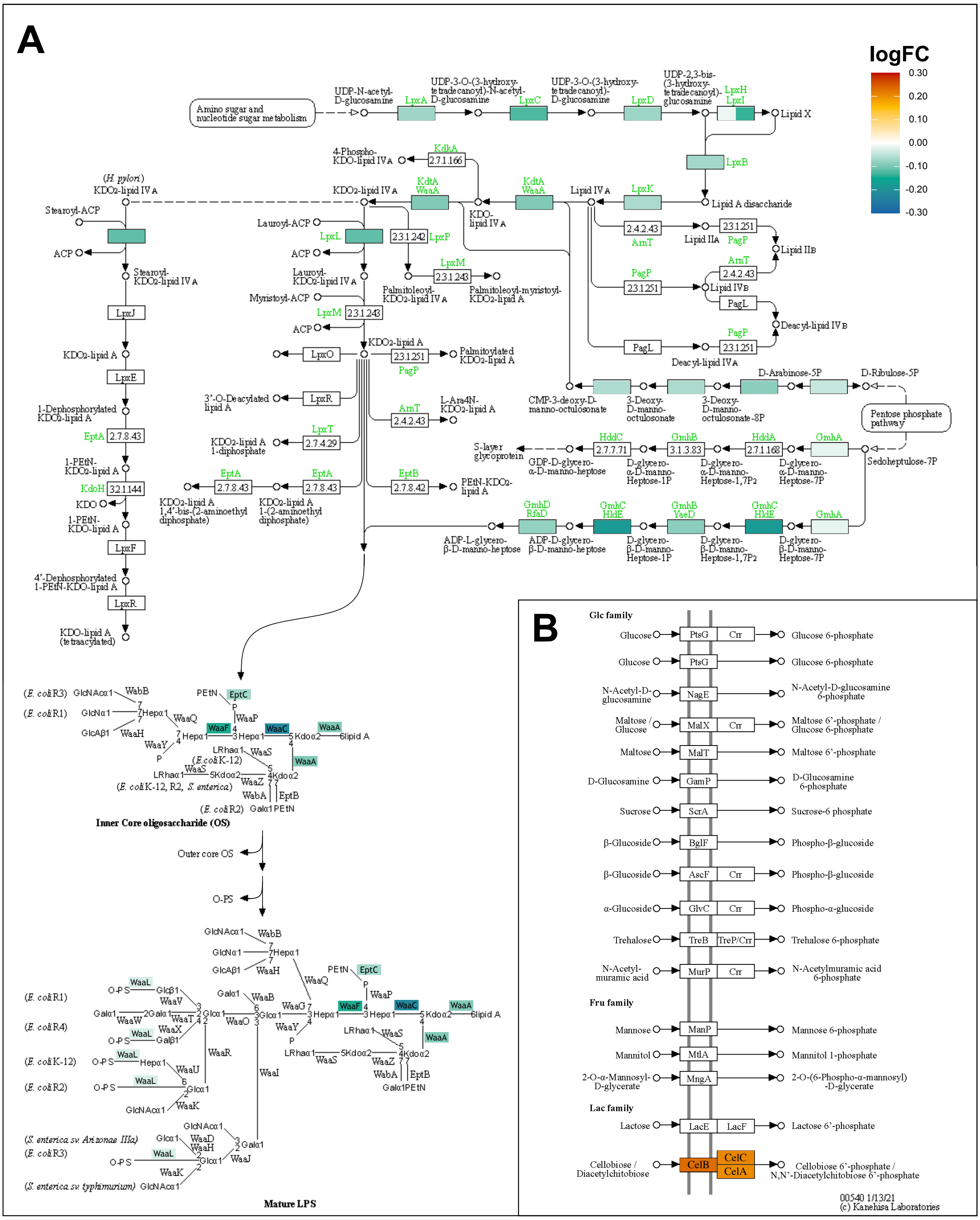

Figure S2: Impact of BEP supplementation on lipopolysaccharide biosynthesis and phosphotransferase system pathways.

(A) The lipopolysaccharide (LPS) biosynthesis pathway (KEGG map00540[2]) in maternal samples during the third trimester of pregnancy.

(B) A subsection of the phosphotransferase system (PTS) pathway (KEGG map02060[2]) in infant samples at 5-6 months of age.

Genes are colored according to $\log_2$FC in abundance, with green and blue indicating reduced abundance in the BEP-supplemented group, and yellow indicating increased abundance in the BEP-supplemented group

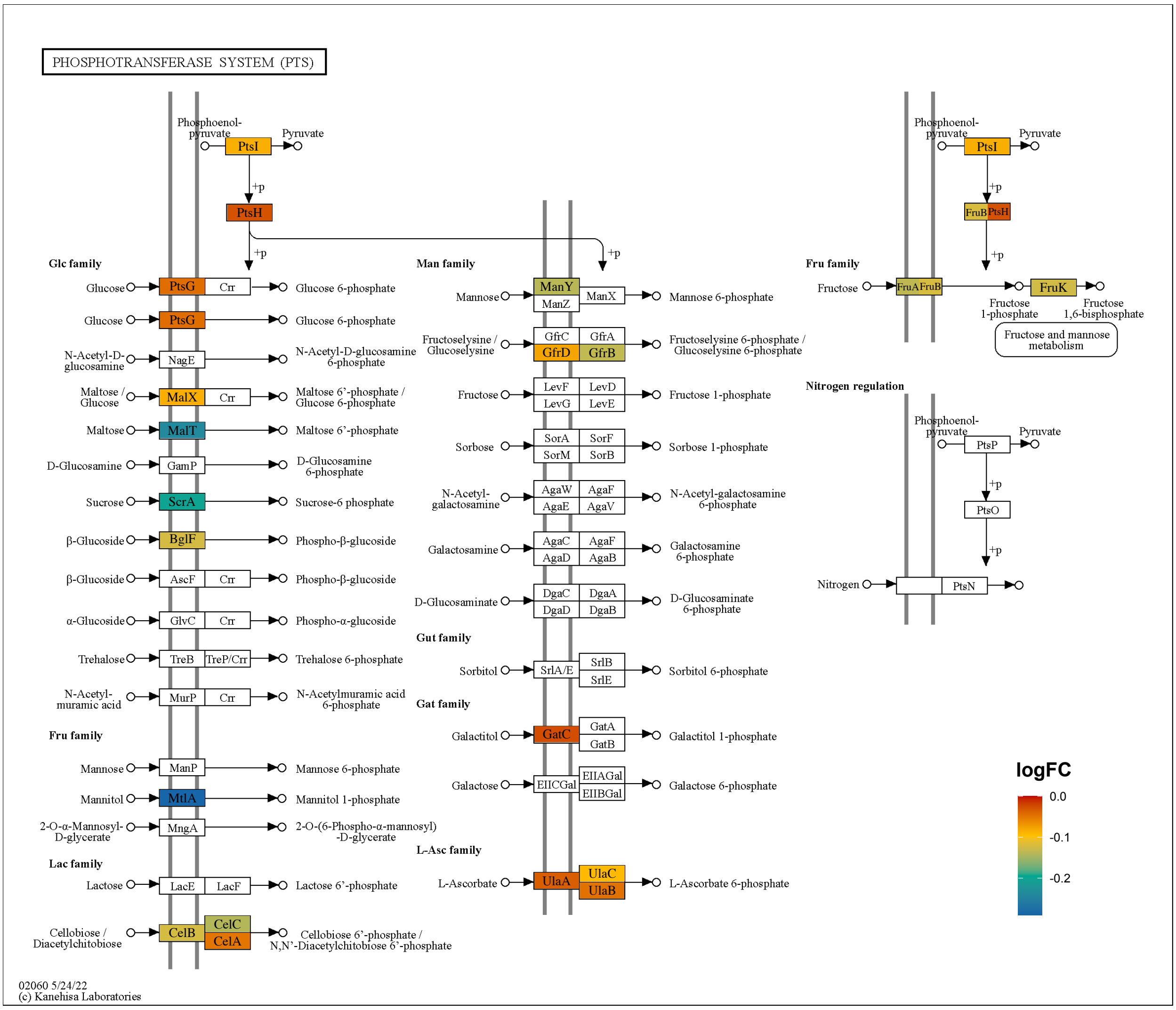

Figure S3: The phosphotransferase system KEGG pathway (map02060[2]) in maternal samples during the third trimester of pregnancy. Genes colored in red, yellow, green, and blue are incrementally less abundantly observed in the BEP-supplemented group.

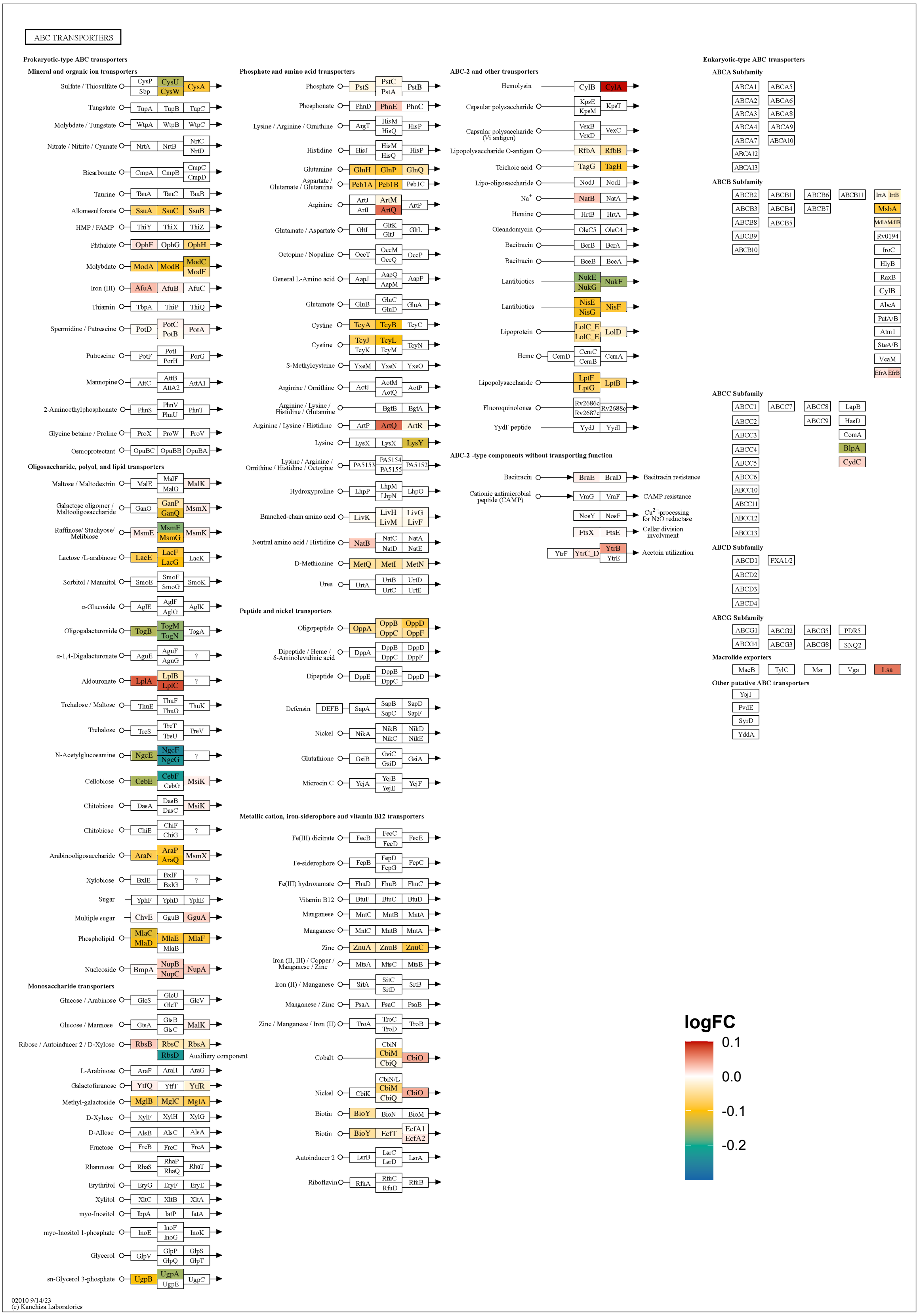

Figure S4: The ABC transporters KEGG pathway (map02010[2]) in maternal samples during the third trimester of pregnancy. Genes colored in yellow, green, and blue are incrementally less abundantly observed in the BEP-supplemented group. Genes colored in red are more abundantly observed in the BEP-supplemented group.

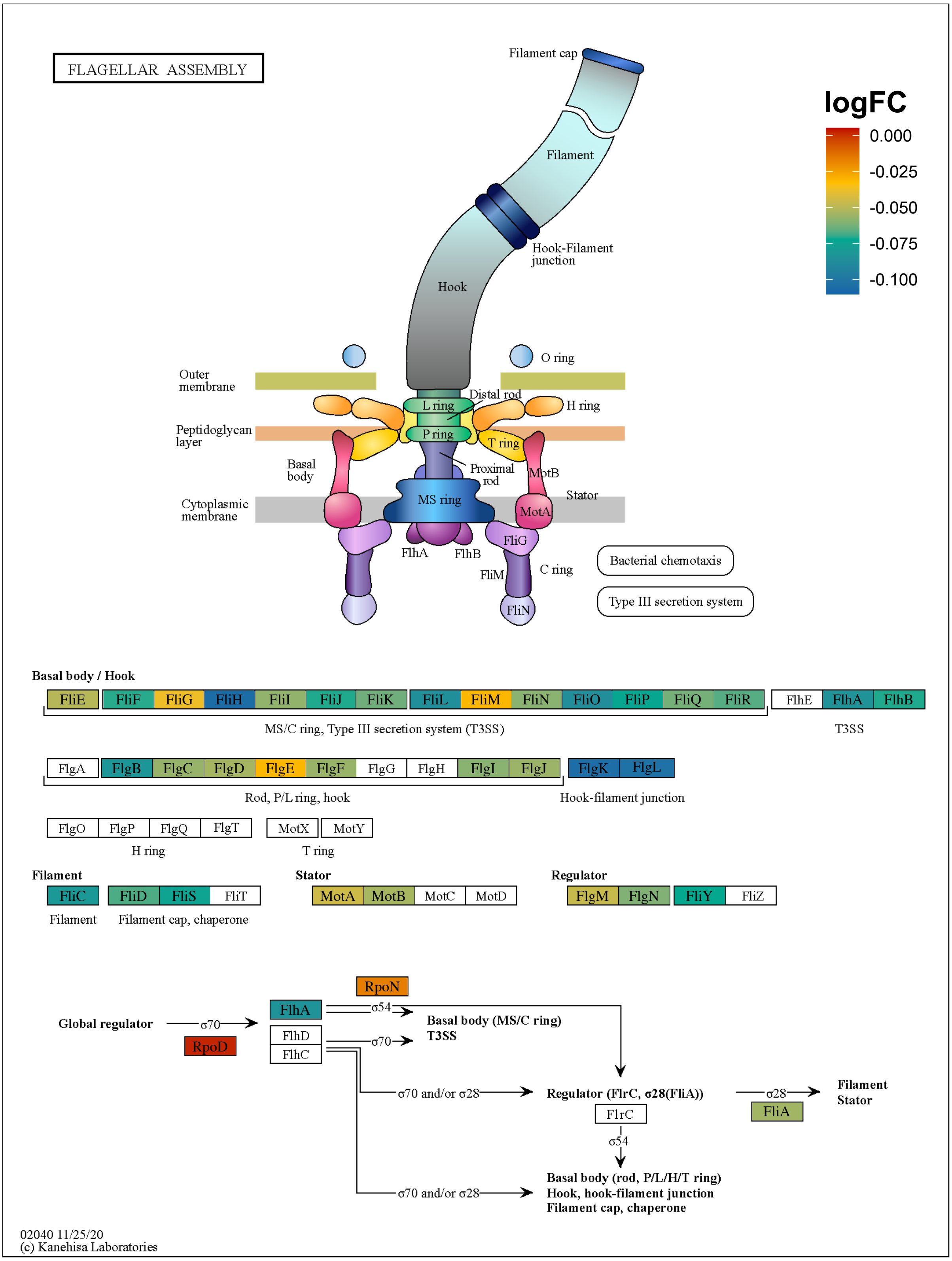

Figure S5: The flagellar assembly KEGG pathway (map02040[2]) in maternal samples during the third trimester of pregnancy. Genes colored in red, yellow, green, and blue are incrementally less abundantly observed in the BEP-supplemented group.

References:

1. Carter, M. M. *et al.* Ultra-deep sequencing of Hadza hunter-gatherers recovers vanishing gut microbes. *Cell* **186**, 3111-3124.e13 (2023).
2. Kanehisa, M. & Goto, S. KEGG: Kyoto Encyclopedia of Genes and Genomes. *Nucleic Acids Research* **28**, 27–30 (2000).
