# Supplementary material for: Maternal balanced energy-protein supplementation reshapes the maternal gut microbiome and enhances carbohydrate metabolism in infants: a randomized controlled trial
Source: Nat Commun. 2025 Mar 18;16:2683. doi: 10.1038/s41467-025-57838-y (PMC11920048; doi:10.1038/s41467-025-57838-y)
Supplement: Supplementary file 2 — Description of Additional Supplementary Files [file 41467_2025_57838_MOESM2_ESM.pdf]

Description of Additional Supplementary Files

File Name: Supplementary Data 1

Description: Results of the differential abundance analyses between intervention groups using ANCOM-BC2, identifying taxa differentially associated with BEP supplementation at each time point and across all time points combined for both maternal and infant samples. ANCOM-BC2 employs a two-sided test, and Benjamini-Hochberg correction is applied to control the false discovery rate. Each sheet corresponds to a single time point, presenting log2 fold change values, adjusted P-values, and significance indicators.

File Name: Supplementary Data 2

Description: Results of the gene set enrichment analyses comparing intervention groups, identifying significantly enriched or depleted KEGG pathways associated with BEP supplementation in mothers and infants across time points. Benjamini-Hochberg correction is applied to control the false discovery rate. Each sheet corresponds to a single time point, providing pathway names, enrichment scores, adjusted P-values, and gene set sizes.

File Name: Supplementary Data 3

Description: Results of the causal mediation analyses assessing the mediating effects of microbiome diversity, individual microbial species, and functional genes on birth outcomes (gestational age, birth weight, and length) and infant anthropometry (WAZ and WLZ at six months). The analyses estimate both the Natural Indirect Effect (NIE) and the Natural Direct Effect (NDE) of maternal and infant microbiome features. Each sheet corresponds to a mediation model, including a series of specific mediators at a given time point for a single outcome, providing effect estimates and 95% confidence intervals for each individual mediator.
