# Supplementary material for: Maternal balanced energy-protein supplementation reshapes the maternal gut microbiome and enhances carbohydrate metabolism in infants: a randomized controlled trial
Source: Nat Commun. 2025 Mar 18;16:2683. doi: 10.1038/s41467-025-57838-y (PMC11920048; doi:10.1038/s41467-025-57838-y)
Supplement: Supplementary file 6 — Reporting Summary [file 41467_2025_57838_MOESM6_ESM.pdf]

Corresponding author(s): *Trenton Dailey-Chwalibóg*

Last updated by author(s): *Feb 5, 2025*

Reporting Summary

Nature Portfolio wishes to improve the reproducibility of the work that we publish. This form provides structure for consistency and transparency in reporting. For further information on Nature Portfolio policies, see our Editorial Policies and the Editorial Policy Checklist.

Statistics

For all statistical analyses, confirm that the following items are present in the figure legend, table legend, main text, or Methods section.

n/a	Confirmed	
☐	☒	The exact sample size (*n*) for each experimental group/condition, given as a discrete number and unit of measurement
☐	☒	A statement on whether measurements were taken from distinct samples or whether the same sample was measured repeatedly
☐	☒	The statistical test(s) used AND whether they are one- or two-sided<br>*Only common tests should be described solely by name; describe more complex techniques in the Methods section.*
☐	☒	A description of all covariates tested
☐	☒	A description of any assumptions or corrections, such as tests of normality and adjustment for multiple comparisons
☐	☒	A full description of the statistical parameters including central tendency (e.g. means) or other basic estimates (e.g. regression coefficient) AND variation (e.g. standard deviation) or associated estimates of uncertainty (e.g. confidence intervals)
☐	☒	For null hypothesis testing, the test statistic (e.g. *F*, *t*, *r*) with confidence intervals, effect sizes, degrees of freedom and *P* value noted<br>*Give P values as exact values whenever suitable.*
☒	☐	For Bayesian analysis, information on the choice of priors and Markov chain Monte Carlo settings
☐	☒	For hierarchical and complex designs, identification of the appropriate level for tests and full reporting of outcomes
☐	☒	Estimates of effect sizes (e.g. Cohen's *d*, Pearson's *r*), indicating how they were calculated

Our web collection on statistics for biologists contains articles on many of the points above.

Software and code

Policy information about availability of computer code

Data collection	*MISAME-III metadata, including infant and maternal anthropometric data, maternal characteristics (level of education, marital status, language, religion, ethnicity, number of jobs, socio-economic status, dietary diversity score and health centers) were collected using SurveySolutions (version 21.5) on tablets by the study physician and midwives. No software was used for stool samples collection.*
Data analysis	*The metagenomic sequencing reads trimming, mapping, and functional profiling were performed using FastP, Bowtie2 and inStrain in Python (version 3.9.15). Taxonomic data analysis and functional data analysis were performed in R (version 4.3.1).*

For manuscripts utilizing custom algorithms or software that are central to the research but not yet described in published literature, software must be made available to editors and reviewers. We strongly encourage code deposition in a community repository (e.g. GitHub). See the Nature Portfolio guidelines for submitting code & software for further information.

Data

Policy information about availability of data

All manuscripts must include a data availability statement. This statement should provide the following information, where applicable:

- Accession codes, unique identifiers, or web links for publicly available datasets
- A description of any restrictions on data availability
- For clinical datasets or third party data, please ensure that the statement adheres to our policy

The metagenomic sequencing data generated in this study have been deposited in the NCBI database under BioProject accession code PRJNA1113165 [https://www.ncbi.nlm.nih.gov/bioproject/PRJNA1113165]. The de-identified personal data are available under restricted access for the purpose of verifying, interpreting, or

extending the research published in this article. Access can be obtained by contacting carl.lachat@ugent.be and signing a data-sharing agreement. The processed taxonomy and gene data are available at https://github.com/stefftaelman/misame-metagenomics-BEP. A response to requests for data access can be expected within 2 months, and the data will be available for one year after signing the data-sharing agreement.

Research involving human participants, their data, or biological material

Policy information about studies with human participants or human data. See also policy information about sex, gender (identity/presentation), and sexual orientation and race, ethnicity and racism.

Reporting on sex and gender	*Our study analyzed metagenomic data from stool samples collected from pregnant and lactating women and their infants. Infants were born during the study phase, and therefore we did not intentionally select infants based on their sex. While we did not perform a sex-specific analysis of infant samples, we collected information on the sex of infants for demographic purposes.*
Reporting on race, ethnicity, or other socially relevant groupings	*Given that our study was conducted in a multi-ethnic country, Burkina Faso, we collected information on the ethnic groupings of our participants to ensure comprehensive reporting and consideration of diversity. The socially relevant categorization variable used in our manuscript is "ethic group." For the purposes of our study, participants were asked to self-identify their ethnic group based on categories provided in the data collection forms. The categories included the major ethnic groups represented in Burkina Faso, such as Bwaba, Mossi and others. We provided clear definitions of these ethnic groups to participants to ensure accurate self-identification.*
Population characteristics	*The average (standard deviation, SD) age of the study participants was 24.3 (5.55) years and body-mass index (BMI) was 22.4 (3.5) kg/m2. The mean (SD) gestational age at birth was 39.8 (1.72) weeks and more than one in three (38.2%) had at least three prior pregnancies. More than half (59.9%) of the women were from food-insecure households and 30.3% were anemic.*
Recruitment	*Women aged between 15 and 40 years and living in the study catchment villages were identified through a census in the study area (n= 10,165). A network of 142 trained village-based project workers visited all eligible women at their homes every 5 weeks to identify pregnancy early, by screening for self-reported amenorrhea. Potential cases were referred to the health center for a urinary pregnancy test. Once gestation was confirmed, the MISAME-III study purpose and procedures were explained in the local languages Mooré, Dioula, or Bwamu. Study eligibility criteria were (i) pregnancy confirmed by a urinary pregnancy test and ultrasound examination; and (ii) written informed consent. Exclusion criteria were (i) gestational age (GA) ≥21 completed weeks; (ii) women who planned to leave the area during their pregnancy or deliver outside the study area; and (iii) women allergic to peanuts.*
Ethics oversight	*MISAME-III has been reviewed and approved by the Commissie voor Medische Ethiek (CME) of Ghent University Hospital (protocol code: B670201734334 and date of 10/08/2020) and the Comité d'Éthique Institutionnel de la Recherche En Sciences de la Santé (CEIRES) of the Institut de Recherche en Sciences de la Santé (IRSS) (protocol code 50-2020/CEIRES and date of 22/10/2020).*

Note that full information on the approval of the study protocol must also be provided in the manuscript.

Field-specific reporting

Please select the one below that is the best fit for your research. If you are not sure, read the appropriate sections before making your selection.

☒ Life sciences ☐ Behavioural & social sciences ☐ Ecological, evolutionary & environmental sciences

For a reference copy of the document with all sections, see nature.com/documents/nr-reporting-summary-flat.pdf

Life sciences study design

All studies must disclose on these points even when the disclosure is negative.

Sample size	*Sample size calculation was indicated in the MISAME-III protocol (https://doi.org/10.1136/bmjopen-2020-038393) and was based on the primary outcome measures of our study: small-for-gestational age (SGA) and length-for-age Z-scores (LAZ) at 6 months of age.*<br>*For the analysis of an effect of the prenatal intervention on SGA, a sample of 652 subjects per prenatal arm is required with α=0.05 and β=0.2. To accommodate for possible loss to follow up, the number of subjects per arm was increased to 888 (total required subjects: 1776). Possible losses are based on previous MISAME studies where the prevalence was ~26 % due to a combination of miscarriage, stillbirths, multifetal pregnancies, outmigration, maternal deaths and incomplete data.*<br>*For the analysis of an effect of the postnatal intervention on LAZ at 6 months of age, the minimum sample size required was 1176 (with α=0.05 and β=0.20). Considering a postnatal loss-to follow-up of 16%, this number was increased to 1400 (i.e., n=1400 singleton live births were required). Considering a possible loss of 26%, this number was further increased to 1891.*<br>*Therefore, a minimum sample size of 1891 will allow us to detect both pre- and postnatal intervention effects.*<br>*A total of 1897 women were enrolled in MISAME-III study. From MISAME-III study, 309 women and their infants from four arms were recruited into Biospé study (https://doi.org/10.3390/nu15184056). Fecal samples from mother-infant dyads were collected in all four arms but only two groups of samples (both pre- and postnatal intervention group and both pre- and postnatal control group, n=152) have been sequenced, which allow us to detect the effect of intervention on gut microbiome.*
Data exclusions	*The following exclusion criteria were described in the MISAME-III protocol: (i) Pregnancies >20 weeks of gestational age. (ii) Women planning on leaving the area during their pregnancy. (iii) Women planning on delivering outside the study area. (iv) Women who are allergic to peanuts. And (v) Women with multifetal gestations (exclusion from analysis).*<br>*In the MISAME-III study, 109 women were excluded due to gestational age ≥21 completed weeks at inclusion (n=59) and multi-fetal*

	pregnancies (n=50).
Replication	*Our research was reported using the Consolidated Standards of Reporting Trials (CONSORT) 2010 checklist. The study design and procedures of the MISAME-III trial were registered on clinicaltrials.gov and thoroughly documented in the protocol available online (https://doi.org/10.1136/bmjopen-2020-038393). Questionnaires used in this study can be found on the misame3.ugent website (https://www.misame3.ugent.be/resources). Laboratory analysis (metagenomic sequencing) for stool samples was conducted using standard methods, as detailed in the Methods section of the current manuscript. Statistical analysis procedures were also documented within the manuscript.*
Randomization	*A stratified permuted block randomization schedule was used to assign women to either the intervention or control arms of the pre- and postnatal interventions. Randomization blocks were generated per health center in blocks of 8 (i.e., 4 controls and 4 interventions arms for each of the pre- and postnatal intervention) using Stata 15.1 (Statacorp, Texas, USA) by a research analyst not involved in the study. The allocation was coded with the letters A(control) or B (intervention) for the prenatal intervention arms, and X (control) and Y (intervention) for the postnatal intervention arms. The allocation letter codes were concealed in sequentially numbered separate, sealed, opaque envelops by the study employees who had no direct contact with the study participants. At enrollment during the first antenatal visit, study midwives assigned particiants to the study groups by drawing the next sealed envelope with the letter code.*
Blinding	*The allocation to intervention or control study group is not blinded from the participants (because the interventions are clearly recognizable). However, outcome assessors will be blinded to the extent possible. The distribution of the study supplements happens during home visits in the villages, while outcome assessment is done at the health center (antenatal consultation, delivery, post-natal growth monitoring). Primary study outcome assessors also have no role in the distribution of the supplements.*

Behavioural & social sciences study design

All studies must disclose on these points even when the disclosure is negative.

Study description	*Briefly describe the study type including whether data are quantitative, qualitative, or mixed-methods (e.g. qualitative cross-sectional, quantitative experimental, mixed-methods case study).*
Research sample	*State the research sample (e.g. Harvard university undergraduates, villagers in rural India) and provide relevant demographic information (e.g. age, sex) and indicate whether the sample is representative. Provide a rationale for the study sample chosen. For studies involving existing datasets, please describe the dataset and source.*
Sampling strategy	*Describe the sampling procedure (e.g. random, snowball, stratified, convenience). Describe the statistical methods that were used to predetermine sample size OR if no sample-size calculation was performed, describe how sample sizes were chosen and provide a rationale for why these sample sizes are sufficient. For qualitative data, please indicate whether data saturation was considered, and what criteria were used to decide that no further sampling was needed.*
Data collection	*Provide details about the data collection procedure, including the instruments or devices used to record the data (e.g. pen and paper, computer, eye tracker, video or audio equipment) whether anyone was present besides the participant(s) and the researcher, and whether the researcher was blind to experimental condition and/or the study hypothesis during data collection.*
Timing	*Indicate the start and stop dates of data collection. If there is a gap between collection periods, state the dates for each sample cohort.*
Data exclusions	*If no data were excluded from the analyses, state so OR if data were excluded, provide the exact number of exclusions and the rationale behind them, indicating whether exclusion criteria were pre-established.*
Non-participation	*State how many participants dropped out/declined participation and the reason(s) given OR provide response rate OR state that no participants dropped out/declined participation.*
Randomization	*If participants were not allocated into experimental groups, state so OR describe how participants were allocated to groups, and if allocation was not random, describe how covariates were controlled.*

Ecological, evolutionary & environmental sciences study design

All studies must disclose on these points even when the disclosure is negative.

Study description	*Briefly describe the study. For quantitative data include treatment factors and interactions, design structure (e.g. factorial, nested, hierarchical), nature and number of experimental units and replicates.*
Research sample	*Describe the research sample (e.g. a group of tagged Passer domesticus, all Stenocereus thurberi within Organ Pipe Cactus National Monument), and provide a rationale for the sample choice. When relevant, describe the organism taxa, source, sex, age range and any manipulations. State what population the sample is meant to represent when applicable. For studies involving existing datasets, describe the data and its source.*
Sampling strategy	*Note the sampling procedure. Describe the statistical methods that were used to predetermine sample size OR if no sample-size calculation was performed, describe how sample sizes were chosen and provide a rationale for why these sample sizes are sufficient.*
Data collection	*Describe the data collection procedure, including who recorded the data and how.*

Timing and spatial scale	*Indicate the start and stop dates of data collection, noting the frequency and periodicity of sampling and providing a rationale for these choices. If there is a gap between collection periods, state the dates for each sample cohort. Specify the spatial scale from which the data are taken*
Data exclusions	*If no data were excluded from the analyses, state so OR if data were excluded, describe the exclusions and the rationale behind them, indicating whether exclusion criteria were pre-established.*
Reproducibility	*Describe the measures taken to verify the reproducibility of experimental findings. For each experiment, note whether any attempts to repeat the experiment failed OR state that all attempts to repeat the experiment were successful.*
Randomization	*Describe how samples/organisms/participants were allocated into groups. If allocation was not random, describe how covariates were controlled. If this is not relevant to your study, explain why.*
Blinding	*Describe the extent of blinding used during data acquisition and analysis. If blinding was not possible, describe why OR explain why blinding was not relevant to your study.*

Did the study involve field work? ☐ Yes ☐ No

Field work, collection and transport

Field conditions	*Describe the study conditions for field work, providing relevant parameters (e.g. temperature, rainfall).*
Location	*State the location of the sampling or experiment, providing relevant parameters (e.g. latitude and longitude, elevation, water depth).*
Access & import/export	*Describe the efforts you have made to access habitats and to collect and import/export your samples in a responsible manner and in compliance with local, national and international laws, noting any permits that were obtained (give the name of the issuing authority, the date of issue, and any identifying information).*
Disturbance	*Describe any disturbance caused by the study and how it was minimized.*

Reporting for specific materials, systems and methods

We require information from authors about some types of materials, experimental systems and methods used in many studies. Here, indicate whether each material, system or method listed is relevant to your study. If you are not sure if a list item applies to your research, read the appropriate section before selecting a response.

Materials & experimental systems

n/a	Involved in the study	
☒	☐	Antibodies
☒	☐	Eukaryotic cell lines
☒	☐	Palaeontology and archaeology
☒	☐	Animals and other organisms
☐	☒	Clinical data
☒	☐	Dual use research of concern
☒	☐	Plants

Methods

n/a	Involved in the study	
☒	☐	ChIP-seq
☒	☐	Flow cytometry
☒	☐	MRI-based neuroimaging

Antibodies

Antibodies used	*Describe all antibodies used in the study; as applicable, provide supplier name, catalog number, clone name, and lot number.*
Validation	*Describe the validation of each primary antibody for the species and application, noting any validation statements on the manufacturer's website, relevant citations, antibody profiles in online databases, or data provided in the manuscript.*

Eukaryotic cell lines

Policy information about cell lines and Sex and Gender in Research

Cell line source(s)	*State the source of each cell line used and the sex of all primary cell lines and cells derived from human participants or vertebrate models.*
Authentication	*Describe the authentication procedures for each cell line used OR declare that none of the cell lines used were authenticated.*

Mycoplasma contamination	*Confirm that all cell lines tested negative for mycoplasma contamination OR describe the results of the testing for mycoplasma contamination OR declare that the cell lines were not tested for mycoplasma contamination.*
Commonly misidentified lines (See ICLAC register)	*Name any commonly misidentified cell lines used in the study and provide a rationale for their use.*

Palaeontology and Archaeology

Specimen provenance	*Provide provenance information for specimens and describe permits that were obtained for the work (including the name of the issuing authority, the date of issue, and any identifying information). Permits should encompass collection and, where applicable, export.*
Specimen deposition	*Indicate where the specimens have been deposited to permit free access by other researchers.*
Dating methods	*If new dates are provided, describe how they were obtained (e.g. collection, storage, sample pretreatment and measurement), where they were obtained (i.e. lab name), the calibration program and the protocol for quality assurance OR state that no new dates are provided.*

☐ Tick this box to confirm that the raw and calibrated dates are available in the paper or in Supplementary Information.

Ethics oversight	*Identify the organization(s) that approved or provided guidance on the study protocol, OR state that no ethical approval or guidance was required and explain why not.*

Note that full information on the approval of the study protocol must also be provided in the manuscript.

Animals and other research organisms

Policy information about studies involving animals; ARRIVE guidelines recommended for reporting animal research, and Sex and Gender in Research

Laboratory animals	*For laboratory animals, report species, strain and age OR state that the study did not involve laboratory animals.*
Wild animals	*Provide details on animals observed in or captured in the field; report species and age where possible. Describe how animals were caught and transported and what happened to captive animals after the study (if killed, explain why and describe method; if released, say where and when) OR state that the study did not involve wild animals.*
Reporting on sex	*Indicate if findings apply to only one sex; describe whether sex was considered in study design, methods used for assigning sex. Provide data disaggregated for sex where this information has been collected in the source data as appropriate; provide overall numbers in this Reporting Summary. Please state if this information has not been collected. Report sex-based analyses where performed, justify reasons for lack of sex-based analysis.*
Field-collected samples	*For laboratory work with field-collected samples, describe all relevant parameters such as housing, maintenance, temperature, photoperiod and end-of-experiment protocol OR state that the study did not involve samples collected from the field.*
Ethics oversight	*Identify the organization(s) that approved or provided guidance on the study protocol, OR state that no ethical approval or guidance was required and explain why not.*

Note that full information on the approval of the study protocol must also be provided in the manuscript.

Clinical data

Policy information about clinical studies
All manuscripts should comply with the ICMJEguidelines for publication of clinical research and a completedCONSORT checklist must be included with all submissions.

Clinical trial registration	*NCT03533712*
Study protocol	*MISAME-III: https://doi.org/10.1136/bmjopen-2020-038393*<br>*Biospé: https://doi.org/10.3390/nu15184056*
Data collection	*This study was conducted in the Houndé health district situated in the Hauts-Bassins region of Burkina Faso. Study inclusion ran from October 2019 to December 2020, and the final child was born on August 7, 2021. Maternal stool samples were collected at inclusion (i.e., the second trimester), the third trimester, 1-2 months postpartum and 5-6 months postpartum. Infant stool samples were collected at 1-2 months and 5-6 months of age corresponding to the maternal sample collection time.*
Outcomes	*The trial has two primary study outcomes that will be used to assess the impact of the prenatal and the postnatal intervention, respectively:*<br>*Incidence of small-for-gestational age (SGA), defined as birth weight <10th centile of the Intergrowth 21st reference.*<br>*Length-for-age z-score (LAZ) calculated using the WHO 2006 growth reference at 6 months of age.*

Dual use research of concern

Policy information about dual use research of concern

Hazards

Could the accidental, deliberate or reckless misuse of agents or technologies generated in the work, or the application of information presented in the manuscript, pose a threat to:

No	Yes	
☐	☐	Public health
☐	☐	National security
☐	☐	Crops and/or livestock
☐	☐	Ecosystems
☐	☐	Any other significant area

Experiments of concern

Does the work involve any of these experiments of concern:

No	Yes	
☐	☐	Demonstrate how to render a vaccine ineffective
☐	☐	Confer resistance to therapeutically useful antibiotics or antiviral agents
☐	☐	Enhance the virulence of a pathogen or render a nonpathogen virulent
☐	☐	Increase transmissibility of a pathogen
☐	☐	Alter the host range of a pathogen
☐	☐	Enable evasion of diagnostic/detection modalities
☐	☐	Enable the weaponization of a biological agent or toxin
☐	☐	Any other potentially harmful combination of experiments and agents

Plants

Seed stocks: *Report on the source of all seed stocks or other plant material used. If applicable, state the seed stock centre and catalogue number. If plant specimens were collected from the field, describe the collection location, date and sampling procedures.*

Novel plant genotypes: *Describe the methods by which all novel plant genotypes were produced. This includes those generated by transgenic approaches, gene editing, chemical/radiation-based mutagenesis and hybridization. For transgenic lines, describe the transformation method, the number of independent lines analyzed and the generation upon which experiments were performed. For gene-edited lines, describe the editor used, the endogenous sequence targeted for editing, the targeting guide RNA sequence (if applicable) and how the editor was applied.*

Authentication: *Describe any authentication procedures for each seed stock used or novel genotype generated. Describe any experiments used to assess the effect of a mutation and, where applicable, how potential secondary effects (e.g. second site T-DNA insertions, mosiacism, off-target gene editing) were examined.*

ChIP-seq

Data deposition

☐ Confirm that both raw and final processed data have been deposited in a public database such as GEO.

☐ Confirm that you have deposited or provided access to graph files (e.g. BED files) for the called peaks.

Data access links
May remain private before publication.: *For "Initial submission" or "Revised version" documents, provide reviewer access links. For your "Final submission" document, provide a link to the deposited data.*

Files in database submission: *Provide a list of all files available in the database submission.*

Genome browser session
(e.g. UCSC): *Provide a link to an anonymized genome browser session for "Initial submission" and "Revised version" documents only, to enable peer review. Write "no longer applicable" for "Final submission" documents.*

Methodology

Replicates

Describe the experimental replicates, specifying number, type and replicate agreement.

Sequencing depth

Describe the sequencing depth for each experiment, providing the total number of reads, uniquely mapped reads, length of reads and whether they were paired- or single-end.

Antibodies

Describe the antibodies used for the ChIP-seq experiments; as applicable, provide supplier name, catalog number, clone name, and lot number.

Peak calling parameters

Specify the command line program and parameters used for read mapping and peak calling, including the ChIP, control and index files used.

Data quality

Describe the methods used to ensure data quality in full detail, including how many peaks are at FDR 5% and above 5-fold enrichment.

Software

Describe the software used to collect and analyze the ChIP-seq data. For custom code that has been deposited into a community repository, provide accession details.

Flow Cytometry

Plots

Confirm that:

- [ ] The axis labels state the marker and fluorochrome used (e.g. CD4-FITC).
- [ ] The axis scales are clearly visible. Include numbers along axes only for bottom left plot of group (a 'group' is an analysis of identical markers).
- [ ] All plots are contour plots with outliers or pseudocolor plots.
- [ ] A numerical value for number of cells or percentage (with statistics) is provided.

Methodology

Sample preparation

Describe the sample preparation, detailing the biological source of the cells and any tissue processing steps used.

Instrument

Identify the instrument used for data collection, specifying make and model number.

Software

Describe the software used to collect and analyze the flow cytometry data. For custom code that has been deposited into a community repository, provide accession details.

Cell population abundance

Describe the abundance of the relevant cell populations within post-sort fractions, providing details on the purity of the samples and how it was determined.

Gating strategy

Describe the gating strategy used for all relevant experiments, specifying the preliminary FSC/SSC gates of the starting cell population, indicating where boundaries between "positive" and "negative" staining cell populations are defined.

- [ ] Tick this box to confirm that a figure exemplifying the gating strategy is provided in the Supplementary Information.

Magnetic resonance imaging

Experimental design

Design type

Indicate task or resting state; event-related or block design.

Design specifications

Specify the number of blocks, trials or experimental units per session and/or subject, and specify the length of each trial or block (if trials are blocked) and interval between trials.

Behavioral performance measures

State number and/or type of variables recorded (e.g. correct button press, response time) and what statistics were used to establish that the subjects were performing the task as expected (e.g. mean, range, and/or standard deviation across subjects).

Acquisition

Imaging type(s) | *Specify: functional, structural, diffusion, perfusion.*

Field strength | *Specify in Tesla*

Sequence & imaging parameters | *Specify the pulse sequence type (gradient echo, spin echo, etc.), imaging type (EPI, spiral, etc.), field of view, matrix size, slice thickness, orientation and TE/TR/flip angle.*

Area of acquisition | *State whether a whole brain scan was used OR define the area of acquisition, describing how the region was determined.*

Diffusion MRI ☐ Used ☐ Not used

Preprocessing

Preprocessing software | *Provide detail on software version and revision number and on specific parameters (model/functions, brain extraction, segmentation, smoothing kernel size, etc.).*

Normalization | *If data were normalized/standardized, describe the approach(es): specify linear or non-linear and define image types used for transformation OR indicate that data were not normalized and explain rationale for lack of normalization.*

Normalization template | *Describe the template used for normalization/transformation, specifying subject space or group standardized space (e.g. original Talairach, MNI305, ICBM152) OR indicate that the data were not normalized.*

Noise and artifact removal | *Describe your procedure(s) for artifact and structured noise removal, specifying motion parameters, tissue signals and physiological signals (heart rate, respiration).*

Volume censoring | *Define your software and/or method and criteria for volume censoring, and state the extent of such censoring.*

Statistical modeling & inference

Model type and settings | *Specify type (mass univariate, multivariate, RSA, predictive, etc.) and describe essential details of the model at the first and second levels (e.g. fixed, random or mixed effects; drift or auto-correlation).*

Effect(s) tested | *Define precise effect in terms of the task or stimulus conditions instead of psychological concepts and indicate whether ANOVA or factorial designs were used.*

Specify type of analysis: ☐ Whole brain ☐ ROI-based ☐ Both

Statistic type for inference | *Specify voxel-wise or cluster-wise and report all relevant parameters for cluster-wise methods.*

(See Eklund et al. 2016)

Correction | *Describe the type of correction and how it is obtained for multiple comparisons (e.g. FWE, FDR, permutation or Monte Carlo).*

Models & analysis

n/a	Involved in the study
☐	☐ Functional and/or effective connectivity
☐	☐ Graph analysis
☐	☐ Multivariate modeling or predictive analysis

Functional and/or effective connectivity | *Report the measures of dependence used and the model details (e.g. Pearson correlation, partial correlation, mutual information).*

Graph analysis | *Report the dependent variable and connectivity measure, specifying weighted graph or binarized graph, subject- or group-level, and the global and/or node summaries used (e.g. clustering coefficient, efficiency, etc.).*

Multivariate modeling and predictive analysis | *Specify independent variables, features extraction and dimension reduction, model, training and evaluation metrics.*
